# Effect of a Fruit and Vegetable Prescription Program on Children’s Fruit and Vegetable Consumption

**DOI:** 10.5888/pcd16.180555

**Published:** 2019-06-13

**Authors:** Ronit A. Ridberg, Janice F. Bell, Kathryn E. Merritt, Diane M. Harris, Heather M. Young, Daniel J. Tancredi

**Affiliations:** 1 Betty Irene Moore School of Nursing, University of California, Davis, Sacramento, California; 2 Center for Healthcare Policy and Research, University of California, Davis, Sacramento, California; 3 Wholesome Wave, Berkeley, California; 4 Division of Nutrition, Physical Activity and Obesity, Centers for Disease Control and Prevention, Atlanta, GA; 5 Department of Pediatrics, University of California, Davis, Sacramento, California

## Abstract

**Introduction:**

Most children in families with low income do not meet dietary guidance on fruit and vegetable consumption. Fruit and vegetable prescription programs improve access to and affordability of health-supporting foods for adults, but their effect on dietary behavior among children is not known. The objective of this study was to describe the extent to which exposure to a fruit and vegetable prescription program was associated with changes in consumption among participants aged 2 to 18.

**Methods:**

We used data from a modified National Cancer Institute screener to calculate fruit and vegetable intake among 883 children who were overweight or had obesity and participated in a 4- to 6-month fruit and vegetable prescription program at federally qualified health centers during 4 years (2012-2015). Secondary analyses in 2017 included paired *t* tests to compare change in fruit and vegetable consumption (cups/day) between first and last visits and multivariable linear regressions, including propensity dose–adjusted models, to model this change as a function of sociodemographic and program-specific covariates, such as number of clinical visits and value of prescription redemption.

**Results:**

We found a dose propensity–adjusted increase of 0.32 cups (95% confidence interval, 0.19–0.45 cups) for each additional visit while holding constant the predicted number of visits and site. An equal portion of the change-score increase was attributed to vegetable consumption and fruit consumption (β = 0.16 for each).

**Conclusion:**

Fruit and vegetable prescription programs in clinical settings may increase fruit and vegetable consumption among children in low-income households. Future research should use a comparison group and consider including qualitative analysis of site-specific barriers and facilitators to success.

**Summary**

**What is already known on this topic?**

Fruit and vegetable prescription programs increase access and affordability of healthy foods among adults, but their effect on children’s dietary behavior is not known.

**What is added by this report?**

Among families with children who were overweight or had obesity (n = 883, 1 child per household) in a 4- to 6-month prescription program at federally qualified health centers, we found a dose propensity–adjusted increase in fruit and vegetable consumption of 0.32 cups per day for each additional visit, with an equal portion attributed to changes in vegetable consumption and fruit consumption.

**What are the implications for public health practice?**

Clinically based fruit and vegetable prescription programs with nutrition education may be an effective way to improve diets for children in low-income households.

## Introduction

In 2007–2010, just 40% of children aged 1 to 18 years met the US Department of Agriculture fruit intake recommendations; 7% met vegetable recommendations (1). Although children’s intake of whole fruit increased significantly from 2003 to 2010, vegetable intake did not change (2). The strongest determinants of fruit and vegetable consumption among young people in the United States are availability/accessibility, taste preferences, parental intake, and race/ethnicity (3,4). Low-income populations have less access to, and lower consumption of, fruits and vegetables (3–5), and diets of children in families using Supplemental Nutrition Assistance Program (SNAP) benefits are less nutritious than diets of children in higher-income families (5).

Healthy food programs at farmers markets and grocery stores provide financial incentives for obtaining fruits and vegetables at the point of purchase (6–9). Incentive studies show that participants increase their daily fruit and vegetable consumption (6) (including specific vegetables [10]), increase their odds of trying new fruits and vegetables (9), and increase their average weekly purchase of fresh fruit (11). In one incentive study, the proportion of participants who reported their diet to be “healthy” or “very healthy” increased from 4% to 63% at 12-month follow-up (12).

Few studies have assessed fruit and vegetable prescription programs, an incentive model that typically includes a “prescription” from a health care provider and nutrition education in a clinical setting. Prescriptions are exchanged for tokens or gift cards or used directly for obtaining fruits and vegetables at participating outlets. Prescription studies among adults at risk for chronic disease demonstrated decreases in hemoglobin A_1c_ levels (13) and body mass index (14) and increases in fruit and vegetable consumption (7). No prescription studies have assessed children’s consumption; only one analyzed data from a pediatric prescription program, but it focused on changes in household food security (15). Our study addressed this gap in knowledge by using data from a pediatric fruit and vegetable prescription program to determine whether the dose of program exposure, specifically, the number of clinical visits and the value of prescription redemption, affected changes in fruit and vegetable consumption over time.

## Methods

Wholesome Wave’s pediatric Fruit and Vegetable Prescription Program (FVRx) was a 4- to 6-month intervention, conducted during 4 years (2012–2015), that targeted change in dietary behavior among children and teenagers aged 2 to 18 years. Health care providers at federally qualified health centers enrolled patient participants who were overweight or had obesity (1 child per household); inclusion criteria consisted of a diagnosis of overweight or obesity based on body mass index (determined by weight-for-age), parental consent, patient willingness to participate, and family intent to make at least 3 program visits. Recruitment methods, including enrollment caps, were determined at the discretion of each site (16). The program provided in-clinic nutrition education and obesity treatment counseling, including guidance on fruit and vegetable consumption and replacement of unhealthy foods with fresh fruits and vegetables, by a physician, nutritionist, and/or trained health educator during monthly clinical visits (16). Participants were permitted a maximum of 6 clinical visits during their enrollment. Providers distributed FVRx prescriptions allocated by household size ($0.50–$1.00/per household member per day) and shared information on times and locations for farmers markets. Participants exchanged prescriptions for coupons or tokens to use directly at farmers market stalls. Details about program design and implementation are described elsewhere (16). This analysis of de-identified data provided by Wholesome Wave was deemed exempt from full review by the University of California, Davis Institutional Review Board.

### Sample

Of 1,269 participants, we included 883 in the analytic sample from 12 clinical sites in Connecticut, Maine, Massachusetts (3), New Mexico, New York (4), Rhode Island, and Washington, DC. We excluded 386 participants for the following reasons: the child was younger than 2 or older than 18 at the first clinical visit (n = 29), we did not have data on fruit and vegetable consumption from the first visit (n = 69), the child had enrolled in a previous year (n = 115), and the child did not attend at least 2 clinical visits or provide at least 2 measurements of fruit and vegetable intake (n = 173).

### Measures

The primary independent variable of program exposure was the number of clinical visits attended by the participant family, and the secondary variable of program exposure was the amount of FVRx redeemed. Data for the dependent variable, recent fruit and vegetable consumption (ie, cups/day), were collected by program providers at each clinical visit by using data from surveys adapted from the National Cancer Institute’s Eating at America’s Table Study Quick Food Scan, which was validated in adults but not in children (17,18). Surveys were administered to parents or caregivers by health care providers or staff members in English or Spanish. Four questions asked about frequency and portion size of fruit and vegetable separately (eg, “Over the last week how many times per day did your child eat vegetables?” or “Each time your child ate vegetables how much did he/she usually eat?”). All fruits and vegetables consumed at mealtimes and snacks (fresh, canned, or frozen) were included, except French fries and fruit juice; individual vegetable types were not specified. Some guidance on portion size from the National Cancer Institute was offered (eg, “about ½ cup” is explained as “half a piece of a medium fruit — for example, half a 6-inch banana or half an orange the size of a tennis ball”). Program facilitators multiplied frequency of consumption by quantity consumed each time to calculate total cups consumed.

We used data on children’s fruit and vegetable consumption (cups/day) at first and last visits to characterize change over time; we further examined fruit and vegetable consumption data against the USDA age- and sex-specific fruit and vegetable consumption recommendations (19) and calculated consumption as a percentage of recommended daily consumption. Farmers market managers collected and recorded redeemed prescriptions and submitted these data to program staff. We calculated the following per household: 1) the average total value of FVRx theoretically prescribed (generated by using an equation based on household size, number of visits, clinical location, and program year), 2) the average total value of FVRx redeemed (using data provided by program staff), and 3) the average proportion of the total value of FVRx theoretically prescribed (dividing the value of FVRx redeemed by value of FVRx theoretically prescribed).

### Statistical analyses

We used Stata software version 13 (StataCorp LLP) for all statistical analyses. We used descriptive statistics (ie, means, medians, standard deviations, range, and proportions) to summarize the distribution of study variables. Paired *t* tests compared daily fruit, vegetable, and combined fruit and vegetable consumption from first to last visits, regardless of the total number of visits, whereas linear regression models examined mean change in fruit and vegetable consumption as a function of sociodemographic and program-dose covariates. Significance was set at *P* < .05.

Unmeasured covariates associated with greater program adherence may have contributed to greater change in fruit and vegetable intake over time, suggesting that unmeasured confounding could account for any dose-response relationship instead of program effectiveness. To adjust for these confounders, we first determined predicted dose propensities for visits and FVRx redemption by using the following covariates associated with visit number and FVRx redemption: participant’s race/ethnicity, sex, and age group; highest level of education of mother or primary caretaker, household size; enrollment in federal food assistance programs (SNAP or Special Supplemental Nutrition Program for Women, Infants, and Children [WIC]), program year, and clinical site. Linear regression models then estimated the dose propensity–adjusted association of FVRx redemption with mean changes in consumption; in this way, we compared changes in consumption along with measures of both expected and actual program exposure (where actual dose is the number of visits attended or prescriptions redeemed, and dose propensity is, on average, the number of visits or FVRx redemption expected from 2 participants who are otherwise similar). We chose to use change scores, as opposed to controlling for baseline intake, because of the large differences at baseline in fruit and vegetable consumption between frequent and infrequent visitors (20). In the regression analyses that compared clinical sites, we used the largest clinical site (Site 11) as the reference site (21). We used robust standard errors for regression parameter estimates to account for residual correlations among participants from the same clinical site and/or heteroscedasticity in residuals.

## Results

The mean age of the 883 participating children was 10 years; most (80%) were younger than 13; more than half (54%) were female (Table 1). Sixty-one percent of the children were Hispanic, followed by non-Hispanic black, African American, or Caribbean American (17%) and non-Hispanic white (17%). About half of participating households had 4 or fewer members; 90% were covered by Medicaid or other public insurance. Sixty-nine percent of households were enrolled in SNAP or WIC. In most (55%) households, the highest education obtained by the participant’s mother or primary caretaker was some high school, a high school degree, or GED.

**Table 1:** Descriptive Characteristics of Participants (n = 883) in Wholesome Wave’s Fruit and Vegetable Prescription (FVRx) Program, 2012–2015^a^

Characteristic	No. (%)^b^
**Total sample (1 child per household)**	883 (100)
**Year of program**	
2012	205 (23)
2013	230 (26)
2014	252 (29)
2015	196 (22)
**Mean (SD) age at enrollment, y**	10 (4)
**Age group at enrollment**	
2 or 3 y	47 (5)
4–8 y	228 (26)
9–13 y	433 (49)
14–18 y	175 (20)
**Female**	480 (54)
**Race/ethnicity**	
Hispanic	540 (61)
Non-Hispanic black, African American, or Caribbean American	148 (17)
Non-Hispanic white	146 (17)
Mixed race or other race	49 (6)
**Household size of ≤4**	453 (51)
**Household health insurance**	
Medicaid/public	795 (90)
Private insurance	66 (7)
Uninsured or other insurance	20 (2)
Missing information	2 (<1)
**Total no. of health clinical visits during program enrollment^c^**	
2	142 (16)
3 or 4	605 (69)
5 or 6	136 (15)
**Mean (SD) number of days between first and last health clinical visit**	87 (32)
**Average value of FVRx prescriptions^d^ redeemed per household, mean (SD), $**	
Average total value of FVRx redeemed per household	361 (230)
Average proportion of redeemed FVRx, of total prescribed	0.59 (0.39)
**Highest level of education of mother or primary caretaker**	
High school classes, high school degree, or GED	490 (55)
Some college or more	233 (26)
Missing information	160 (18)
**Household is enrolled in SNAP or WIC**	608 (69)
**The 3 largest sites, by number of participants enrolled**	
Site 11	181 (21)
Site 4	148 (17)
Site 7	95 (11)

Abbreviations: SD, standard deviation; SNAP, Supplemental Nutrition Assistance Program; WIC, Special Supplemental Nutrition Program for Women, Infants, and Children.

a Data were collected from surveys administered to parents or caregivers by health care providers or staff members in English or Spanish. Participants were children and teenagers aged 2 to 18 years who were overweight or had obesity.

b All values are number (percentage) unless otherwise indicated. Percentages may not sum to 100 because of rounding.

c Participants were allowed a maximum of 6 clinical visits.

d Participants exchanged prescriptions for coupons or tokens to use directly at farmers market stalls.

Average household redemption of FVRx prescriptions during the program was $361. Most participants (69%) made 3 or 4 clinical visits over an average period of 87 (standard deviation, 32) days in a single program year during their enrollment. Average FVRx redemption proportion was 59%; 9% of families exceeded the theoretical maximum for redemption. Three of the 12 clinical sites enrolled almost half of all participants (49%). Approximately one-quarter of participant data were collected from each program year, with slightly more from 2014 (29%).

Mean daily consumption at first visit was 1.6 cups of fruit, 1.2 cups of vegetables, and a combined 2.8 cups of fruits and vegetables (Table 2); daily means were larger by an average of 0.1 to 0.3 cups at last visit (fruits, 1.7 cups; vegetables, 1.3 cups; fruits and vegetables combined, 3.1 cups). These amounts corresponded to an increase from first to last visit in the percentage of federal dietary guidelines being met of 93% to 100% for fruits, 64% to 70% for vegetables, and 78% to 86% for combined fruits and vegetables. Participants with 5 or 6 visits consumed about 25% more combined fruits and vegetables at baseline than their counterparts with just 2 visits. In unadjusted paired *t* tests, the mean change in combined daily fruit and vegetable consumption from first visit to last visit was 0.26 (95% confidence interval [CI], 0.13–0.39) cups. Similarly, linear regression models adjusted for sociodemographic and program-related covariates showed a significant dose-response increase of fruit and vegetable consumption of 0.32 cups per additional clinical visit (95% CI, 0.20–0.45; *P* < .001) (Table 3). We found no significant dose response for the secondary independent variable of total FVRx redemption.

**Table 2:** Fruit and Vegetable Consumption at First Visit and Last Visit and Change in Overall Consumption Among Participants (n = 883) in Wholesome Wave’s Fruit and Vegetable Prescription Program, 2012–2015^a^

Consumption	First Visit		Last Visit		Change Between First and Last Visits, No. of Cups, Mean (SD) [95% CI]^b^
	No. of Cups, Mean (SD)	Percentage of Dietary Guidelines	No. of Cups, Mean (SD)	Percentage of Dietary Guidelines	
Daily fruit consumption	1.6 (1.3)	93	1.7 (1.3)	100	0.13 (1.2) [0.05–0.21]
Daily vegetable consumption	1.2 (1.1)	64	1.3 (1.2)	70	0.13 (1.1) [0.06–0.21]
Daily combined fruit and vegetable consumption	2.8 (2.2)	78	3.1 (2.1)	86	0.26 (2.0) [0.13–0.39]

Abbreviations: CI, confidence interval; SD, standard deviation.

a Data were collected from surveys administered to parents or caregivers by health care providers or staff members in English or Spanish. Participants were children and teenagers aged 2 to 18 years who were overweight or had obesity.

b Change in consumption between first and last visits was determined by unadjusted paired *t* tests. All *P* values < .001.

**Table 3:** Regression Coefficients for Change in Combined Fruit and Vegetable Consumption Among Participants in Wholesome Wave’s Fruit and Vegetable Prescription Program, 2012–2015^a^

Model and Unit of Measure	Adjusted for Sociodemographic and Program Covariates, β (95% CI)	Adjusted for Dose Propensity Scores, β (95% CI)
**Model 1: No. of Visits (n = 723 Participants)**		
**Change in fruit and vegetable consumption per 1 additional clinical visit**	0.32 (0.20 to 0.45)^b^	0.32 (0.19 to 0.45)^b^
**Predicted number of visits^c^**	—	−0.18 (−0.92 to 0.57)
**Race/ethnicity**		
Hispanic	Reference	
Non-Hispanic black, African American, or Caribbean American	−0.03 (−0.61 to 0.56)	—
Non-Hispanic white	−0.40 (−0.86 to 0.06)	—
Mixed race or other race	−0.98 (−1.72 to −0.22)	—
**Sex**		
Female	Reference	—
Male	−0.12 (−0.17 to 0.41)	—
**Age group, y**		
2 or 3	Reference	—
4–8	0.29 (−0.35 to 0.89)	—
9–13	0.58 (−0.28 to 1.19)	—
14–18	0.66 (−0.02 to 1.34)	—
**Highest level of education of mother or primary caretaker**		
High school classes, high school degree, GED, or missing information	Reference	—
Some college or more	0.28 (−0.04 to 0.59)	—
**Program year**		
2012	0.21 (−0.27 to 0.68)	—
2013	−0.34 (−0.79 to 0.11)	—
2014	Reference	—
2015	0.20 (−0.29 to 0.69)	—
**Household size**	0.06 (−0.03 to 0.16)	—
**Enrollment in SNAP or WIC**		
No	Reference	—
Yes	0.17 (−0.14 to 0.48)	—
**Clinical site**		
Site 1	−0.09 (−0.61 to 0.44)	0.13 (−0.42 to 0.67)
Site 2	−0.68 (−1.53 to 0.17)	−0.27 (−1.21 to 0.67)
Site 3	0.52 (−0.19 to 1.23)	0.71 (0.04 to 1.39)^d^
Site 4	0.03 (−0.58 to 0.63)	0.17 (−0.32 to 0.65)
Site 5	−0.23 (−0.95 to 0.48)	0.25 (−0.51 to 1.01)
Site 6	0.49 (−0.31 to 1.29)	0.86 (0.07 to 1.65)^d^
Site 7	0.30 (−0.44 to 1.04)	0.36 (−0.13 to 0.86)
Site 8	0.42 (−0.28 to 1.11)	0.26 (−0.37 to 0.89)
Site 9	0.44 (−0.33 to 1.21)	0.25 (−0.47 to 0.98)
Site 10	0.74 (0.15 to 1.34)^d^	0.73 (−0.03 to 1.49)
Site 11	Reference	—
Site 12	−0.03 (−0.99 to 0.93)	−0.11 (−1.11 to 0.89)
**Model 2: FVRx Redemption^e^ (n = 673 Participants)**		
**Actual redemption per $100 of redemption**	0.08 (−0.01 to 0.18)	0.08 (−0.02 to 0.18)
**Predicted redemption^f^**	—	0.02 (−0.14 to 0.17)
**Race/ethnicity**		
Hispanic	Reference	—
Non-Hispanic black, African American, or Caribbean American	0.04 (−0.1 to 0.18)	—
Non-Hispanic white	−0.38 (−0.88 to 0.11)	—
Mixed race or other race	−0.95 (−1.78 to −0.12)^d^	—
**Sex**		
Female	Reference	—
Male	0.16 (−0.16 to 0.47)	—
**Age group, y**		
2 or 3	Reference	—
4–8	0.34 (−0.36 to 1.04)	—
9–13	0.69 (0.01 to 1.37)	—
14–18	0.68 (−0.07 to 1.43)	—
**Highest level of education of mother or primary caretaker**		
High school classes, high school degree, GED, or missing information	Reference	—
Some college or more	0.25 (−0.09 to 0.59)	—
**Program year**		
2012	0.22 (−0.29 to 0.72)	—
2013	−0.33 (−0.79 to 0.12)	—
2014	Reference	—
2015	0.12 (−0.56 to 0.80)	—
**Household size**	0.01 (−0.12 to 0.14)	—
**Enrollment in SNAP or WIC**		
No	Reference	
Yes	0.18 (−0.15 to 0.51)	—
**Clinical site**		
Site 1	0.17 (−0.37 to 0.72)	0.38 (−0.50 to 1.27)
Site 2	−0.86 (−1.74 to 0.02)	−0.38 (−1.35 to 0.59)
Site 3	0.52 (−0.28 to 1.32)	0.60 (−0.11 to 1.30)
Site 4	0.15 (−0.51 to 0.80)	0.16 (−0.30 to 0.61)
Site 5	−0.23 (−0.95 to 0.49)	0.17 (−0.61 to 0.95)
Site 6	0.41 (−0.43 to 1.25)	0.79 (−0.03 to 1.60)
Site 7	0.26 (−0.54 to 1.05)	0.29 (−0.23 to 0.81)
Site 8	0.59 (−0.16 to 1.24)	0.43 (−0.17 to 1.03)
Site 9	0.57 (−0.21 to 1.34)	0.24 (−0.40 to 0.89)
Site 10	0.93 (0.29 to 1.57)^g^	0.81 (0.17 to 1.46)^d^
Site 11	Reference	Reference
Site 12	−0.06 (−1.10 to 0.99)	−0.35 (−1.31 to 0.62)

Abbreviations: CI, confidence interval; SNAP, Supplemental Nutrition Assistance Program; WIC, Special Supplemental Nutrition Program for Women, Infants, and Children.

a Data were collected from surveys administered to parents or caregivers by health care providers or staff members in English or Spanish. Participants were children and teenagers aged 2 to 18 years who were overweight or had obesity.

b *P* < .001.

c The number of visits we would expect families/participants to have, on the basis of their characteristics.

d *P* < .05.

e Participants exchanged prescriptions for coupons or tokens to use directly at farmers market stalls.

f The redemption propensity score we would expect families/participants to have, on the basis of their characteristics.

g *P* < .01.

In dose propensity–adjusted regression models holding constant the predicted number of visits and clinical site (Table 3), the association of each additional clinical visit with the primary outcome of mean change in fruit and vegetable consumption was nearly a third of a cup (β = 0.32; 95% CI, 0.19–0.45). An equal portion of that increase was attributed to vegetable consumption (β = 0.16; 95% CI, 0.08–0.23) and fruit consumption (β = 0.16; 95% CI, 0.08–0.25). We found no significant relationship between the FVRx redemption and mean consumption. In the propensity-adjusted models, the coefficients for 3 clinical sites were significantly higher than the coefficient for the reference site for combined daily fruit and vegetable consumption, by more than a half-cup (Site 3, β = 0.71; Site 6, β = 0.86; Site 10, β = 0.81) (Table 3).

## Discussion

Using causal inference methods to account for measured and unmeasured confounders, we found a significant and meaningful change in children’s fruit and vegetable consumption associated with dose of program exposure (number of visits). We found a significant increase of nearly a third of a cup in mean fruit and vegetable consumption, which, despite programmatic and study differences, was greater than the overall total increase in fruit and vegetable consumption (nearly a quarter-cup) among participants in a large-scale nutrition incentive study, which focused on adults (6). The propensity dose–adjusted findings suggest an even more critical outcome: for 2 participants otherwise similar, the one with an additional clinical visit added 0.32 cups to their combined fruit and vegetable consumption. Recommended daily fruit and vegetable consumption for moderately active children and teenagers ranges from 2 cups for girls aged 2 or 3 to 5 cups for boys aged 14 to 18 (2); the effect of a third-cup or half-cup increase in fruit and vegetable intake on overall dietary intake and quality varies, but it is meaningful at any age and increases the proportion of recommended total fruit and vegetable consumption (22).

In systematic reviews of nutrition interventions for children aged 12 or younger, most increases in consumption are attributed to increases in fruit and less so in vegetables (23). Similarly, recent changes in children’s dietary intake nationally reveal increases primarily in fruit consumption (2). Our study demonstrated significant increases in vegetable consumption equal to increases in fruit consumption. The program’s educational component emphasized vegetables, in addition to healthier choices, such as replacing fruit juice with fruit (written communication, Skye Cornell, Chief Programs Officer, Wholesome Wave; April 2018). Perhaps more pointedly, this increase in vegetables could be due to availability of offerings at farmers markets, which are of higher quality (fresher) and more diverse (uncommon cultivars/heirloom varieties) (24). Because more than 90% of American children do not meet federal guidelines on vegetable consumption, this unique finding is important and warrants further inquiry.

Perhaps surprising was the lack of association between increased total prescription redemption and increased fruit and vegetable consumption, suggesting a possible substitution effect as families reallocated spending and limiting an anticipated long-term effect of such a program. The clinical encounter and/or nutrition education may have had a greater influence on behavior change than the financial incentive. This possibility is consistent with a 2016 review of 33 price interventions promoting healthy dietary behavior among adults, which found that the most effective programs at increasing consumption included nutrition education (25).

Differences in food access (eg, number and location of participating markets, desirability of produce selection) or capacity (eg, budgets, staff time, technical assistance) among sites may also have affected increases in fruit and vegetable consumption, consistent with a previous analysis of a subset of this FVRx data set (15). The site with the greatest increase in consumption (Site 10) was considered a strong program site overall, having an established partnership with the local farmers market before FVRx implementation, expanded hours, and a year-round market season (Skye Cornell, written communication, April 2018). Although programs and data collection across sites were mostly consistent (typically during May–October), a year-round market may indicate greater awareness and acceptance among the community. Within-site homogeneity of children’s race/ethnicity precludes comparisons of the overall findings by race/ethnicity. However, the racial/ethnic make-up of the 3 sites with significant increases in fruit and vegetable consumption differed: at Site 10, 100% (n = 47) of the participating children were non-Hispanic white; at Site 6, 100% (n = 27) were Hispanic, and at Site 3, 90% (n = 42) were Hispanic. These data suggest that the FVRx program is culturally adaptable.

Study strengths include accounting for possible self-selection bias and control for some measured and unmeasured confounders by using change scores and causal inference methods, specifically dose propensity–adjusted regression models. In addition, the study capitalized on a large data set from a novel program operationalized in a clinical setting. Program implementation under real-world conditions, rather than in a controlled trial, has helped the institutional operators iterate program design over the years. For example, concerns over limited access to farmers markets led to a partnership with a national retail chain in 2017 and 2018 to increase shopping convenience and possibly overall FVRx redemption. Although our results may have limited generalizability, outcomes from this real-world natural experiment offer proof of concept to help lay groundwork for a prospective trial.

Our study had several limitations. We relied on self-reported dietary intake. Nutrition interventions that rely on self-reported dietary intake are often limited by design, but they can inform dietary guidance and public health policy (26). Reliability and validity tests of the National Cancer Institute’s fruit and vegetable screeners suggest underestimation of intake for both adult men and women (27), but they have not been tested with children. However, parental proxy reporting can adequately assess a range of food and nutrient intakes among children and may be more accurate when completed in collaboration with the child (28).

Our sample’s average baseline consumption of fruits and vegetables was higher than national rates, particularly for lower-income households (29); on the other hand, our screener excluded 100% fruit juice from fruit intake estimates, while national data often include it. Our sample’s higher intake may indicate self-selection bias, if families and children predisposed to healthier consumption patterns were also predisposed to enroll in this program. Nevertheless, the same screener was used for all clinical visits, so the change between visits merits attention. Additionally, even if consumption levels were inflated for the group as a whole, they were still below recommended levels and thus well-suited to intervention. Although we could not compare the results of those who completed the program with the results of those who dropped out (those who lacked 2 measurements of fruit and vegetable intake), we did conduct a sensitivity analysis by including those with one visit in the regression analyses and assuming no fruit and vegetable change, and we found no substantive change in our findings. Finally, our self-reported data may also have been influenced by a disproportionate inclusion of persons with low health literacy (30).

Despite these limitations, our findings support growing interest in collaborative partnerships between the health care sector, farmers markets, and community organizations, to address poor diet and, by extension, overall health. With a growing number of prescription-based incentive programs now at the local, city, and state level (supported with $25 million for a federal pilot program in the 2018 Farm Bill), it is critical to conduct more rigorous studies to determine best practices to achieve highest impact, including tests of geographic location, retail type (market vs grocery store), and food-type restrictions. Because most prescription programs rely on grant funding, which may be unsustainable, more evidence on the cost-effectiveness of the FVRx intervention for chronic disease prevention is warranted before health care organizations or insurers will agree to integrate it into their care portfolios. In addition, studies using true experimental design with complementary fruit and vegetable assessment tools, such as 24-hour recall, could bolster the scientific evidence base. Overall, more consistent use of selected indicators or measures across prescription programs could improve the ability to assess outcomes and improve program design on a larger scale.
